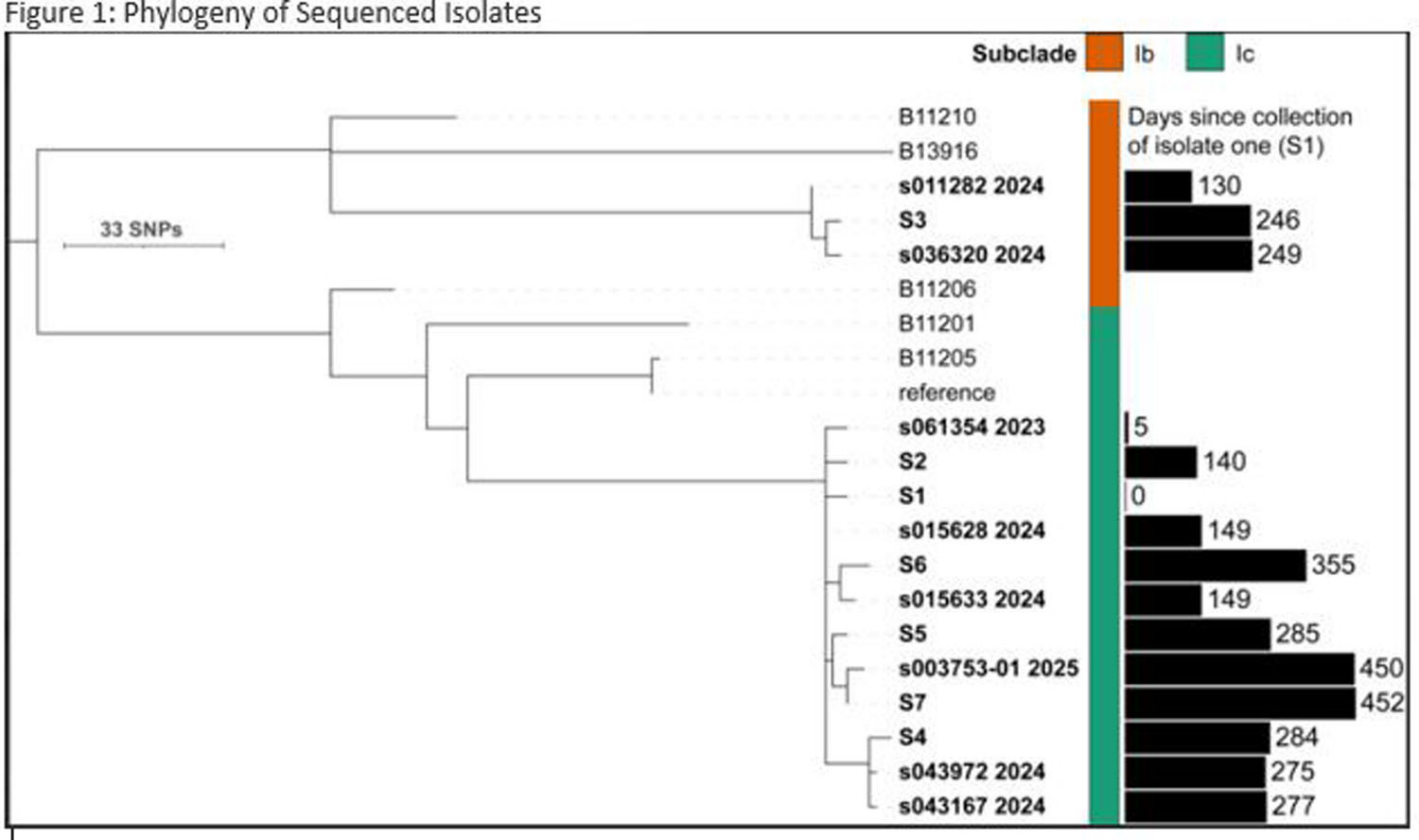# 339 Weekly Antibiotic Time-Outs Improve Stewardship Without Compromising Safety on a High-Use Medical Unit

**DOI:** 10.1017/ash.2026.10680

**Published:** 2026-06-23

**Authors:** Preeti Mehrotra, Ashley Dauphin, Nicholas Cauldron, Christina Cuomo, Patrick Gordon, Dana Pepe, Matthew Lee, James Kirby, Sharon Wright

**Affiliations:** 1 Bidmc; 2 Beth Israel Deaconess Medical Center; 3 Brown University; 4 Beth Israel Lahey Health

## Abstract

**Background:**?The incidence of Candidozyma auris (C. auris) is growing in healthcare settings internationally. International healthcare exposure, compromised skin integrity, lengthy intensive care unit stays all increase risk of C. auris colonization. In Massachusetts, clusters and outbreaks related to C. auris have mostly been characterized by transmission in long term care settings. Over the course of 1.5 years, our institution experienced a cluster of C. auris cases initially hypothesized to be linked to one long stay index patient. Method:?At our tertiary academic medical center, one long stay patient with international healthcare exposure developed a hospital-onset C. auris bloodstream infection. In accordance with public health guidance, any time a clinical isolate is identified, point prevalence screening (PPS) is conducted on impacted unit(s). When possible, we also attempted to proactively screen patients with known recent international healthcare exposure for C. auris, though this was not implemented systematically. Given the protracted nature of our cluster and significant overlap in inpatient spaces and time of several patients, we used Nanopore and Illumina genomic sequencing to identify chains of transmission and to inform infection prevention related interventions. Result:?Over the course of the we identified clinical isolates, positive PPS isolates, and positive screening isolates for patients with international healthcare exposure, for a total of 19 isolates. Of these 19 isolates, genomic sequencing was performed on 15 isolates. Genomic analysis revealed three separate introductions of C. auris into our facility, representing subclades Ib, Ic, and III. The largest cluster involving 1 isolates (7 clinical, 4 PPS) comprised of subclade Ic was related to the long stay index patient. The second cluster involved isolates from subclade Ib and was related to a patient who was screened pro-actively for international healthcare exposure. **Conclusion:** In healthcare settings where C. auris is an emerging but not yet an endemic threat, genomic sequencing can play a critical role in understanding transmission events. At our institution, discovering multiple introductions of C. auris into our facility was unexpected. Genomic sequencing provided reassurance in our infection control interventions and demonstrated the need for serious consideration of a targeted pro-active screening program.

**Figure:**